# Influence of Paired Pandemic of COVID-19 and HIV Infection on Pregnant Women and Children: a Challenging Issue

**DOI:** 10.34763/jmotherandchild.20212502.d-21-00017

**Published:** 2022-04-01

**Authors:** Naina Kumar, Mishu Mangla

**Affiliations:** 1 Department of Obstetrics and Gynaecology, All India Institute of Medical Sciences, Bibinagar, Hyderabad Metropolitan Region, Telangana, India

**Keywords:** Children, coronavirus disease 2019, human immunodeficiency virus, pandemic, pregnancy

## Abstract

COVID-19 infection started in China in December 2019 and was declared a global pandemic of international concern by the WHO in March 2020. With rapid spread of infection worldwide, health systems and health care programs came to a standstill, leaving essential services such as antenatal care and human immunodeficiency virus (HIV) comprehensive care for children and pregnant women completely devastated. Furthermore, due to lockdowns, children and pregnant women living with HIV were forced to stay at home with no access to health facilities, loss of follow-up, and discontinuation of antiretroviral drugs therapy. The present review briefs concerning the impact of COVID-19 and HIV/AIDS pandemics on children and pregnant women worldwide.

## Introduction

The COVID-19 pandemic, which started in December 2019 from the city of Wuhan, China, has affected the health services of almost every country worldwide, resulting in numerous short- and long-term adverse outcomes. The pandemic has reversed the progress of many national and international programs meant for human uplift. One such significant impact of the COVID-19 pandemic is on the comprehensive services provided to combat human immunodeficiency virus (HIV) infections in pregnant women and children. The pandemic has severely affected HIV prevention, treatment, and care services, making many people, especially adolescents, young women, and children, vulnerable to its consequences. The data relating to the risk of acquiring COVID-19 infection and its progress in pregnant women and children living with HIV is minimal. However, an increased risk might be due to the immunocompromised state in these patients. Furthermore, pregnant women with HIV infection are at greater risk of rapid deterioration with COVID-19 due to physiological or psychological vulnerability [1]. The present manuscript goes over some of the major challenges faced by countries in slowing the progress of HIV/AIDS and COVID-19, which have emerged as the biggest paired pandemic of the century.

## Material and methods

The recent literature related to pregnant women and children living with HIV/AIDS was searched from various governmental agencies including WHO, UNICEF, UNAIDS, Global Funds, and English peer-reviewed journals from databases like PubMed, Scopus, Google Scholar, EMBASE, and others. The search terms used were: COVID and HIV; HIV/AIDS in children and pregnant women during COVID; Children and HIV/AIDS.

## Current global scenario of HIV/AIDS

India alone accounts for 23.49 lakh (2,349,000) people living with HIV/AIDS and ranks third in the countries most affected by HIV/AIDS. In this challenging era of COVID-19, when most of the resources are pushed towards the management of COVID-19 infection, the lives of HIV-infected immunocompromised pregnant women and children are severely affected. They are now struggling with several pandemics together: COVID-19, fear and anxiety, lack of access to health care services, lack of food, and lack of antiretroviral treatment services [2]. According to a recent UNICEF report, it was found that the pandemic-related disruptions in health services have threatened to reverse the hard-won progress achieved for children and pregnant women in their struggle against HIV/AIDS. It was observed that globally an estimated 2.8 million children and adolescents and around 1.3 million pregnant women living with HIV have suffered from the adverse effects of disrupted health services due to COVID-19–related restrictive measures [3]. Furthermore, the parents of an additional 13.8 million children have succumbed to death from HIV and AIDS-related complications due to lack of services, making these children prone to suffering [3]. In addition to this, according to recent UNAIDS data, it was found that around 880 children and adolescents get infected with HIV, and 310 die from AIDS-related causes every day, revealing a massive burden of HIV/AIDS on this vulnerable group of the population [4].

## Disrupted HIV prevention, testing, and treatment services during the pandemic

As a result of restrictive measures announced to control the COVID-19 pandemic, the health system globally is shattered. There is a disruption in services necessary to prevent transmission of HIV infection from mother to child and treatment by antiretroviral therapy (ART), resulting in an escalating rise in new cases. It has been projected that the number of new HIV infection cases will double if 100% of the population is deterred from accessing treatment services, and similar results will be seen with pediatric deaths from HIV infections. Hence, if the lockdown and restrictive measures continue for the next six months, there will be only 770,000 children and pregnant women able to receive life-saving treatment to prevent onward HIV infection [3]. This will result in a rise in HIV-related mortality by 10% in the coming five years, especially in low-and middle-income countries which bear the maximum burden of the disease [5]. Furthermore, a recent study from sub-Saharan Africa has reported that the COVID-19 pandemic has severely affected health services dealing with HIV prevention, testing, and treatment of new patients, though the treatment of people already living with HIV/AIDS and on ART remained almost unaffected till June 2020 [6].

One major step to prevent HIV transmission from mother to child is by early testing and the start of treatment. The COVID-19 pandemic has also affected the HIV testing services significantly, resulting in increased fear of vertical transmission as a smaller number of antenatal women are undergoing HIV testing in their early pregnancies [7]. A recent survey reported that the COVID-19 pandemic has severely reduced pregnant women’s access to HIV testing and treatment in 13 important countries by 25% to 50% since the beginning of the pandemic [4]. Moreover, a global survey of approximately 500 health facilities in Africa and Asia reported that due to COVID-19– related lockdowns and restrictions between April and September 2020, the antenatal visits of pregnant women dropped drastically by 5% in sub-Saharan Africa and by around 66% in many Asian countries, resulting in missed occasions for early HIV testing and initiation of treatment in pregnant women, thereby increasing the chances of mother-to-child transmission and a rise in the number of children born with HIV infection [8]. Furthermore, with the declaration of the COVID-19 outbreak as a pandemic, the health care workers involved in the care of people living with HIV were diverted towards COVID-19 patients, the HIV clinics were converted to COVID-19 isolation wards, and the HIV testing platforms to COVID-19 testing labs, making the critical services of diagnostic and viral load testing in pregnant and lactating women and for children inaccessible [9]. Moreover, the funds which were previously being used to fight against the global threat of HIV/AIDS are now being diverted towards the management of COVID-19.

According to recent WHO figures, an estimated 27.5 million people living with HIV globally were receiving ART in 2020, accounting for a 73% coverage rate. In children and adolescents, which constitute a vulnerable group, the estimates of ART coverage are significantly lower (54%), indicating the need to scale up efforts to protect this high-risk population [10]. In addition to this, the COVID-19 pandemic has also affected the initiation of treatment in newly diagnosed children with HIV infection, with a recorded fall of 25% to 50% [4]. Even before the onset of the pandemic, only 59% of HIV-exposed children globally were tested by two months of age, and only 53% were reported to be taking antiretroviral medications [11,12]. With the onset of the pandemic, these figures are expected to go down further. In the past 10 years (from 2010 to 2019), ART coverage among pregnant women with HIV has almost doubled, from 45% to 85%, but this progress was halted by the pandemic, making the longstanding dream of eliminating mother-to-child transmission of HIV out of reach in the near future [4].

## The way forward

The best way to prevent transmission of HIV infection from mother to child is through early antenatal testing. Hence, promoting HIV testing at community levels, hospitals, peripheral centers, and hotspots will help in the early detection of antenatal cases, which can be put on ART as soon as they are diagnosed, to reduce the risk of transmission to the baby. Promoting HIV self-testing can also be very useful in the current scenario of the pandemic, when people, especially pregnant women, are not able to visit health care facilities due to fear of COVID-19 and lockdown restrictions. It is a convenient and confidential mode of HIV testing and can help in maintaining HIV testing rates during the pandemic [13,14]. Furthermore, health care centers, satellite clinics, and support for health workers should be strengthened, so that children, adolescents, and pregnant women who have lost follow-up during the pandemic can have access to HIV services and care. Identification of at-risk children, adolescents, and young women, testing for HIV, and initiation of antiretroviral drug therapy will all help in controlling the HIV epidemic during this pandemic. In addition to this, pregnant women and new mothers living with HIV should be provided comprehensive maternal and newborn care, HIV services, and treatment, postpartum contraception advice, and access to the full range of modern contraceptives. To prevent and control HIV infection in children, early infant diagnosis as recommended by WHO should be started at 6–8 weeks of age in all infants delivered to women with HIV infection or those at risk using point-of-care diagnostics for early diagnosis and management. Adolescents and young women who constitute a vulnerable group, especially during the pandemic period due to increased risk of sexual exploitation, should be offered testing and care in high-risk areas. Awareness programs should be conducted among adolescent girls about HIV prevention, transmission, and care, contraception, its use and availability, and safe sexual practices. Furthermore, recent guidelines mention that people living with HIV/AIDS, including pregnant women, should receive SARS-CoV-2 vaccines, irrespective of their CD4 or viral load, as it was observed that the potential benefits of the vaccine outweigh the risks [15].

## Formulating policies and guidelines

Every country, especially those where HIV/AIDS is prevalent, should formulate policies to slow down the progress of both COVID-19 and HIV/AIDS in the vulnerable group, including pregnant women, adolescents, and children. A recent study has reported that national ministries of health in sub-Saharan Africa have improved HIV service delivery guidelines, to safeguard continuous access to ART and restrict contact with health facilities. They have developed and expedited a differentiated service delivery approach for HIV treatment which is a patient-centered approach with services adapting to cater to the needs and expectations of the patients [6]. Furthermore, extending access to ART refills, or multi-month dispensing, will help in reducing the number of clinical consultations, thereby eliminating the fear of contracting COVID-19 infection among people on ART, without affecting their HIV/AIDS treatment during the pandemic era [6]. In addition to this, since pregnant women are more prone to acquire HIV infections, they require effective prevention measures in many countries. Such women should be offered pre-exposure counseling and prophylaxis to protect them and their newborns from the stigma of HIV infection [16]. In India also the risk of HIV infection in adolescents and pregnant women is high; currently, indigenous HIV self-testing is not available in India, but the health ministries are working hard to make the HIV self-test approach an integral part of the National AIDS Control Program (NACP), especially during pandemics [17].

## Conclusion

The COVID-19 pandemic has halted many essential health care services, including HIV prevention, testing, and treatment. It has imposed serious challenges for children, adolescents, and pregnant women living with HIV worldwide. After one and a half years of the COVID-19 pandemic, lockdowns, restrictions, quarantines, and disruptions of health care services, the progress of many global goals, including elimination of AIDS among children by 2030, has been severely affected, but the dream is important and still achievable. Addressing pregnant women and children living with HIV should be made a priority by providing them critical care including antiretroviral therapy and testing of neonates delivered to infected mothers. Adolescent girls, pregnant women, and children constitute the most vulnerable group during the paired pandemic of HIV and COVID-19 and hence, should be taken care of by optimising funding and strengthening the health care system at all levels.

## Key points

- The COVID-19 pandemic has severely affected pregnant women and children living with HIV/AIDS.
- Essential services, including HIV prevention, testing, and treatment, have come to a halt during the pandemic.
- Early antenatal testing for HIV has dramatically fallen in low- and middle-income countries.
- Risk of mother-to-child transmission may increase during the pandemic.
